# Visualization of Glutamate Decarboxylase Activity in Barley Seeds under Salinity Stress Using Mass Microscope

**DOI:** 10.3390/metabo12121262

**Published:** 2022-12-14

**Authors:** Soichiro Ikuta, Eiichiro Fukusaki, Shuichi Shimma

**Affiliations:** 1 Department of Biotechnology, Graduate School of Engineering, Osaka University, 2-1 Yamadaoka, Suita, Osaka 5650871, Japan; 2 Osaka University Shimadzu Omics Innovation Research Laboratory, Osaka University, 2-1 Yamadaoka, Suita, Osaka 5650871, Japan; 3 Institute for Open and Transdisciplinary Research Initiatives, Osaka University, 2-1 Yamadaoka, Suita, Osaka 5650871, Japan

**Keywords:** glutamate decarboxylase activity, barley seeds, salinity stress, mass spectrometry imaging, γ-aminobutyric acid, seed germination

## Abstract

γ-Aminobutyric acid (GABA) accumulates in plants in response to environmental stresses. The activity levels of glutamate decarboxylase (GAD), an enzyme involved in GABA biosynthesis, are reported to increase during germination under salinity stress. However, it is not clear which tissues of the plant seeds are affected by GAD activity in response to salinity stress. In this study, the effects of salinity stress on the distribution of barley seeds GAD activity during germination were investigated. The mass spectrometry imaging (MSI) method was optimized, and the distribution of GAD activity in germinated seeds exposed to salinity stress at different germination stages from 12 to 48 h after imbibition was investigated. In this study, MSI was successfully applied to enzyme histochemistry to visualize the relative GAD activity in germinating barley seeds for the first time. The salinity stress increased the GAD activity, mostly due to the increase in relative GAD activity in the embryo. Higher GAD activity was detected in seeds exposed to salinity stress in the scutellum or aleurone layer, which are difficult to separate for extraction. This method can be used to clarify the role of GABA shunts, including GAD enzyme responses, in barley seeds under stress.

## 1. Introduction

Seed germination is an important stage in plant growth and development. It is also an important aspect of agriculture because it affects the crop yield and quality [1]. Soil salinity affects plant germination through osmotic stress and ion toxicity and has a significant effect on the yield of various crops worldwide [2]. Therefore, an understanding of how plant seeds respond to and overcome salinity stress, which leads to germination, is extremely important. In plants, γ-aminobutyric acid (GABA) acts as an adaptive metabolite that accumulates in response to various environmental stimuli, such as low and high temperatures, drought, hypoxia, and mechanical injury [3]. However, the GABA metabolic pathway may play a role in the stress response and the associated signaling [4]. Glutamate decarboxylase (GAD, EC 4.1.1.15) catalyzes the reaction to produce GABA from glutamate by decarboxylation. As an enzyme involved in GABA biosynthesis, GAD also plays an important role in stress responses, for example in soybean seeds [5,6], fava bean seeds [7,8], and kidney bean seeds [9]; moreover, salinity and osmotic stresses are reported to increase GAD activity levels and GAD gene expression during seed germination. However, it remains unclear which tissues of germinating seeds are affected by GAD activity in response to salinity stress.

Mass spectrometry imaging (MSI) is used to visualize the distribution of compounds [10]. This technique can be used to visually localize a target compound directly on a tissue section via direct tissue ionization. Enzyme histochemistry using MSI has recently attracted attention as a method for visual enzyme activity localization. As with conventional enzyme histochemistry, this technique involves the reaction of substrates catalyzed by enzymes present in the tissue sections, followed by the detection and visual localization of the products. Using this method, the substrate and product can be detected simultaneously. This technique has been used for the visual localization of cholinesterase [11] and choline acetyltransferase [12], and dipeptidyl peptidase B [13]. Furthermore, we successfully visualized the relative activity of GAD in legume seeds during the germination stage using enzyme histochemistry and MSI [14] and applied this method to investigate the effect of salinity stress on the localization of GAD activity in germinating plant seeds.

Barley seeds are a widely used model plant species for seed germination studies. Barley is the fourth highest produced cereal crop in the world [15] and an important cereal grain. Its germinated seeds contain more GABA than its ungerminated seeds [16] and are used as GABA-rich food. Barley is relatively tolerant to salinity stress and can grow in conditions of 150 to 500 mM NaCl [17]. Therefore, it has been widely used in salinity stress response studies [18,19]. However, the effect of salinity stress on GAD enzyme activity and the localization of this enzyme activity in barley seeds have not been investigated.

The objective of this study was to investigate the applicability of MSI-based enzyme histochemistry to investigate the effects of salinity stress on the localization of site-specific GAD activity in germinating barley seeds. First, the effects of salinity stress on GAD activity at different germination stages of barley seeds were investigated using LC/MS analysis. Next, the enzymatic reaction conditions were investigated to visually localize GAD in germinating barley seeds using MSI analysis. The localization of GAD activity in salinity-stressed seeds was compared with that in control seeds at several germination stages. The effect of salinity stress on the localization of GAD activity in the seed embryo and the aleurone layer was further investigated using high-resolution analysis. This paper is the first successful visualization of the localization of GAD activity in barley seed and the first report of the effect of salinity stress on the localization of GAD activity in plant seeds.

## 2. Experimental Design

### 2.1. Chemicals and Reagents

L-Glutamic acid-^15^N, the substrate for the enzyme reaction, was purchased from Sigma-Aldrich (St. Louis, MO, USA), and L-Glutamic-2,4,4-d3 acid (Glu-d3) from the CDN isotope (Pointe-Claire, QC, Canada). The internal standard, *d*_4_-DL-alanine, was purchased from Santa Cruz Biotechnology (Dallas, TX, USA). The matrix, α-cyano-4-hydroxycinnamic acid (CHCA), was purchased from Merck (Darmstadt, Germany). The ethylenediaminetetraacetic acid disodium salt (EDTA) added to the substrate buffer for the enzyme reaction was purchased from Dojindo Laboratories (Kumamoto, Japan), and pyridoxal 5′-phosphate (PLP) acetonitrile, used as the LC/MS mobile phase, was purchased from Kanto Chemical Co. (Tokyo, Japan), ethanol and trifluoroacetic acid were purchased from Dojindo Laboratories (Kumamoto, Japan), dipotassium hydrogen phosphate was purchased from Nacalai Tesque (Kyoto, Japan), and potassium dihydrogen phosphate was purchased from Fujifilm Wako Pure Chemicals (Osaka, Japan). Ultrapure water was taken from a Genpure UV-TOC × CAD PLUS system (Thermo Fisher Scientific, Waltham, MA, USA).

### 2.2. Plant Growth Conditions

Barley seeds (*Hordeum vulgare*, var. Shinjuboshi) were purchased from Kaneko Seeds Co., Ltd. (Gunma, Japan). For seed germination, paper tissue (Prowipe, Daio Paper Corporation, Tokyo, Japan) was placed on a Petri dish (Propio Petri dish 100 mm × 100 mm × 15 mm, As One, Osaka, Japan) and moistened with 10 mL of ultrapure water or 100 mM NaCl solution to mimic exposure to salt stress. This concentration was selected because it was the highest concentration at which barley seeds germinated at a high rate in a previous study [19]. Barley seeds were sown on moistened beds and kept in the dark for germination. Seeds were collected 12, 24, 36, and 48 h after sowing and frozen at –80 °C until analysis.

### 2.3. Measurement of GAD Activity Using LC/MS

Five germinated barley seeds for each germination stage and for each treatment (ultrapure water and NaCl solution) were placed in 50 mL tubes for freezing and grinding (Yasui Kikai, Osaka, Japan) in the presence or absence of salt stress at each germination stage. The tubes were frozen in liquid nitrogen, placed in grinding metal (Yasui Kikai, Osaka, Japan), cooled with liquid nitrogen, and ground to a powder (2000 rpm, 5 s) using a multi-bead shocker (Yasui Kikai, Osaka, Japan). The tubes were then frozen in liquid nitrogen to prevent dissolution and 30 mg of each sample was weighed (*n* = 3). Then, 500 μL of buffer (phosphate buffer: 70 mM, pH 5.8; EDTA: 2 mM; and PLP: 0.2 mM) was added to each sample, and a shaking incubator (25 °C, 30 min, 1200 rpm) was used to prepare a crude enzyme solution. The supernatant was then collected by centrifugation (4 °C, 10,000 rpm, 10 min). The enzyme reaction was performed by mixing 80 μL of the supernatant with 40 μL of 50 mM Glu-^15^N solution and incubating at 40 °C for 30 min. To stop the enzymatic reaction, the supernatant was incubated at 90 °C for 10 min. After centrifugation (4 °C, 10,000 rpm, and 10 min), 100 μL of the supernatant was collected and 300 μL of methanol and 20 μL of internal standard (20 μmol/mL *d*_4_-DL-alanine solution in 50% MeOHaq) were added to separate the lipid components. The protein was then removed using ultrapure water and chloroform and diluted 5-fold using the mobile phase solution. One hundred microliters were dispensed into a vial with a 250 µL insert and analyzed. LCMS-8060 (Shimadzu Corporation, Kyoto, Japan) coupled to a Nexera HPLC System (Shimadzu Corporation) was used for the analysis. A crownpak CR-I (+) column (3.0 mm i.d., 150 mm, 5 mm; Daicel CPI, Osaka, Japan) was used for chromatographic separation. The relative GAD activity was determined by calculating the area of the GABA-^15^N peak divided by the area of the *d*_4_-DL-alanine peak.

### 2.4. Creating Sections of Barley Seeds

For section preparation, barley germinated seeds were placed in molds (Base mold A, Falma, Japan), mounted in 4% carboxymethylcellulose solution, and then frozen at −80 °C. Frozen sections were prepared using a cryostat (CM1950, Leica Biosystems, Wetzlar, Germany) at −20 °C. The sections were acquired using cryofilm (Section-Lab, Hiroshima, Japan). The sample sections were fixed on ITO (Indium Tin Oxide) glass slides (Matsunami, Osaka, Japan) with conductive double-sided tape (3M, Tokyo, Japan). Three different sections were prepared for Opitimization of the reaction conditions. For further visualization of relative GAD activity in the embryo and periphery of the seed coat, four sections were prepared from two salt-stressed barley seeds and two control barley seeds.

### 2.5. On-Tissue Enzyme Reaction

The GAD substrate solution was prepared as a mixture of the below final concentrations: 70 mM phosphate buffer (pH 3.0, 5.8, and 8.0), 2 mM EDTA, 0.2 mM PLP 50 mM Glu-d3. An airbrush (GSI Creos, Tokyo, Japan) was used to supply 50 μL of GAD substrate solution per section to the barley section. The sections were placed in a heated chamber at 40–60 °C for the enzymatic reaction.

### 2.6. MALDI-MSI Analysis

The matrix, CHCA, was evaporated on the ITO glass with tissue sections using a vacuum evaporation system (iMLayer; Shimadzu Corporation, Kyoto, Japan) at 250 °C for 3 min to assist ionization. Matrix-assisted laser desorption/ionization mass spectrometry (MALDI-MSI) analysis was performed using an iMScope QT (Shimadzu Corporation). The mass spectra were obtained in the *m/z* 95–155 range in the positive ion detection mode. The analysis software Imagereveal MS (Shimadzu Corporation) was used to obtain MS images.

### 2.7. Statistical Analysis

To visualize the relative GAD activity, the maximum GABA-d3 intensity was set to 100%, and the percentage of GABA-d3 values at all analyzed points in the sections was expressed as the relative GAD activity. Region of interest (ROI) analysis was performed on the relative GAD enzyme activity images using Imagereveal MS. Bar graphs were generated based on the mean relative GAD activity values obtained from eight ROIs. Student’s *t*-test was used to test for significant differences between the means of two paired groups, ANOVA was used to test for significant differences between the means of three or more groups, and *t*-tests were employed for post hoc analysis. The Bonferroni method was used for correction.

## 3. Results and Discussion

### 3.1. Determination of GAD Activity Using LC/MS

**Figure 1:**
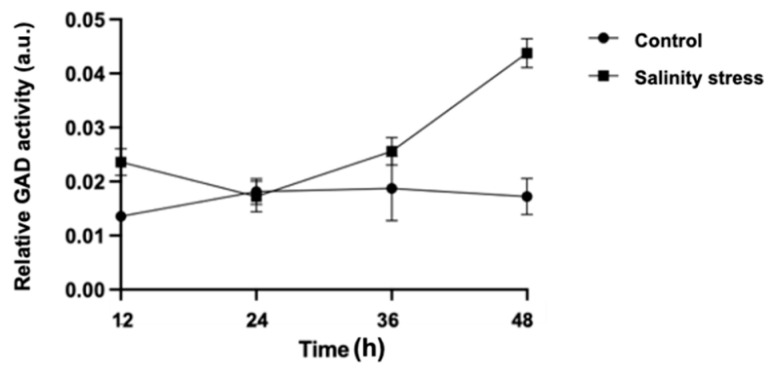
Changes in relative GAD activity during barley seed germination with or without salinity stress.

To investigate the effect of salinity stress on GAD activity in germinated barley seeds, the relative GAD activity in germinated barley seeds at 12, 24, 36, and 48 h after imbibition treatment was measured using LC/MS (Figure 1). The results reveal that the relative GAD activity of the control seeds remained almost unchanged during germination, whereas that of the seeds exposed to salinity stress tended to increase. Furthermore, when compared at each germination stage, the relative GAD activity was significantly higher in the salinity-stressed germinated seeds than in the control seeds at 12, 36, and 48 h (Figure S1). In a previous study, the responses of the GAD expression genes in three barley seed varieties (Athrouh, Acsad175, and Rum) to salinity stress were investigated, and the results reveal that salinity stress increased the quantity of mRNA encoding the GAD protein [19]. In this study, the GAD activity may have increased because of increased GAD expression. However, GAD is a calmodulin-dependent enzyme that catalyzes GABA synthesis from Glu in the cytosol and transports it to the mitochondria [20]. In plants, increased calcium ion concentrations have been reported to increase GABA concentrations by activating GAD [21]. Therefore, the salinity stress may also affect the post-translational regulation of GAD proteins in germinating barley seeds.

### 3.2. Construction of a Method for the Visualization of GAD Activity in Barley Seeds

**Figure 2:**
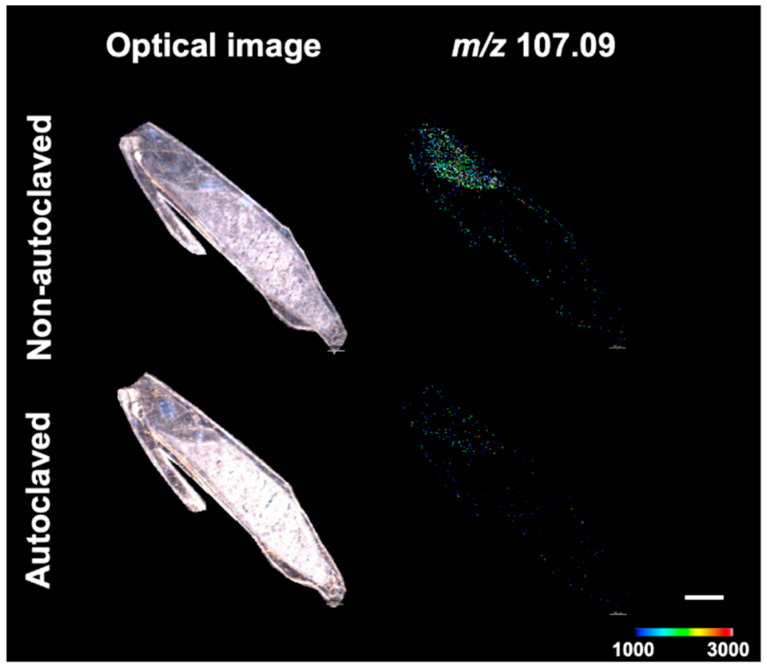
Confirmation of substrate conversion by GAD in germinating barley seeds. Optical image and GABA-d3 distribution (*m/z* 107.09) of non-autoclaved and autoclaved seed section. Scale bar is 1 mm.

LC-MS analysis revealed that salinity stress tended to increase GAD activity in germinated barley seeds. Therefore, the GAD activity in germinated barley seeds exposed to salinity stress was localized by enzyme histochemistry and MSI to identify the tissues in which GAD increased. To establish a method for the visual localization of GAD activity in germinated barley seeds, the enzymatic conversion of Glu-d3 to GABA-d3 by GAD was confirmed in germinated barley seed sections. The substrate Glu-d3 was supplied by airbrush, followed by incubation at 40 °C for 15 min. As in a previous study [14], *m*/*z* 107.09 was selected as the peak derived from GABA-d3. To further confirm that GABA-d3 was produced by GAD in barley seed sections, the same experiment was performed on autoclaved sections to inactivate the enzyme. GABA-d3 was detected in the non-autoclaved seed sections but not in the autoclaved sections (Figure 2). These results confirmed the enzymatic reaction of Glu-d3 to GABA-d3 by GAD in germinated barley seed sections.

**Figure 3:**
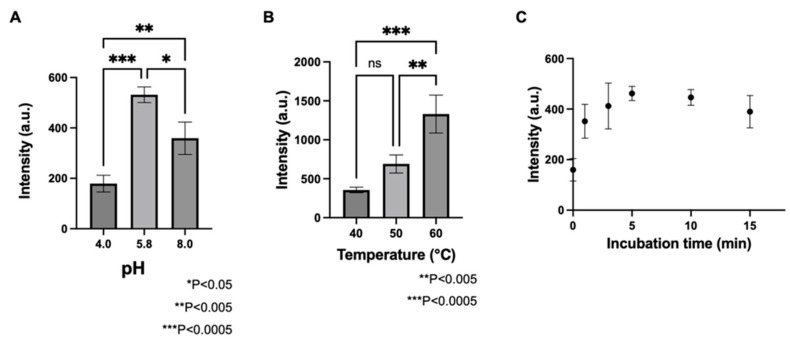
Comparison of GABA-d3 intensity (**A**) under different pH levels; (**B**) at different reaction temperatures, and (**C**) for different reaction times. *n* = 3.

Next, the pH of the substrate solvent, enzyme reaction temperature, and enzyme reaction time were optimized for the visual localization of GAD activity. The results showed that the activity was significantly stronger at pH 5.8 than at pH 3.0 and 8.0 (Figure 3A). In a previous study, the optimum pH for GAD in barley seeds was found to be approximately 5.0–6.0, which is consistent with the present results [22]. Therefore, a pH of 5.8 was selected for the substrate solvent in this study.

Enzyme reaction temperatures of 40 °C, 50 °C, and 60 °C were compared. The activity was significantly higher at 60 °C (Figure 3B). Therefore, 60 °C was selected as the enzyme reaction temperature in this study. This optimum temperature has also been reported for germinated rice seeds [23].

The average intensity of GABA-d3 detected at different incubation times between 0–15 min is shown in Figure 3C. In this study, the intensity of GABA-d3 detection at an incubation time of 3 min was considered representative of the distribution of GAD activity relative to GAD enzyme activity, as the reaction proceeded linearly.

### 3.3. Visualization of GAD Activity during Germination under Salinity Stress

**Figure 4:**
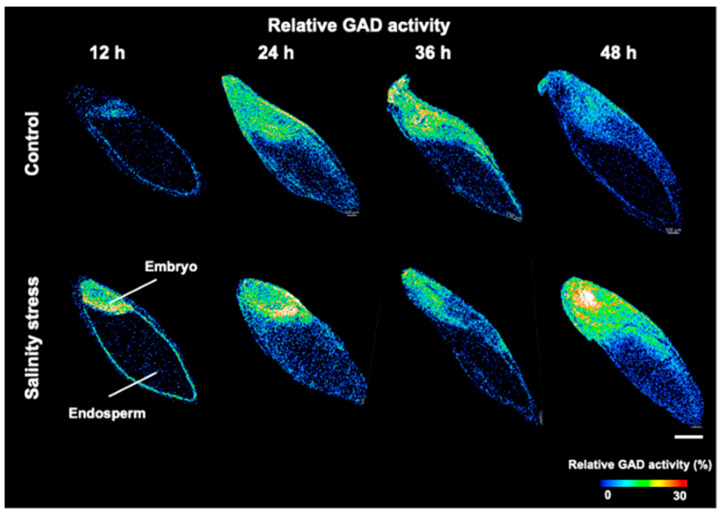
Relative glutamate decarboxylase (GAD) activity distributions during barley seed germination with or without salinity stress. Scale bar is 1 mm.

Using optimized enzymatic reaction conditions, barley seeds at different germination stages were exposed to salinity stress to visualize the relative GAD activity (Figure 4). In addition, ROI analysis was used to compare the relative GAD activity of the embryo and the endosperm. The relative GAD activity was higher in the embryo than in the endosperm of barley seeds exposed to salinity stress at all germination stages (Figure S2). According to recent reports, the GABA shunt pathway, a bypass pathway of the tricarboxylic acid (TCA) cycle, is activated in response to various environmental stressors such as cold, high temperature, and drought [4].

In the GABA shunt pathway, glutamate dehydrogenase metabolizes ammonia to 2-oxoglutarate to produce glutamate, and GAD then produces GABA and CO_2_. GABA is then converted to succinate via succinate-semialdehyde by GABA-T, which then joins the TCA cycle. Salinity stress is known to enhance the GABA shunt pathway [4], and increased activities of GAD and GABA shunt have been reported in soybean seeds exposed to salinity stress [24]. The GABA shunt activity is affected by GAD activity, which is dependent on pH and calcium ion levels [25]. Furthermore, the accumulation of reactive oxygen species (ROS) is increased in plant tissues under environmental stress conditions, including salinity stress, and the enhancement of the GABA shunt pathway effectively suppresses the generation of ROS [26]. Therefore, antioxidants may play an important role in protecting germinating embryos and in achieving successful germination. The presence of antioxidant flavonoids increases in germinating barley seed embryos upon salinity stress [27], suggesting that increased GAD activity and GABA shunting in the embryo may be involved in the antioxidant-mediated salinity stress response. Therefore, the increased GAD activity in germinating barley seed embryos may respond to antioxidant-mediated salinity stress by enhancing the GABA shunt pathway. To confirm this, the metabolites, enzymes, and antioxidants involved in the GABA shunt pathway require investigation.

Additionally, GAD activity was detected in the endosperm. The endosperm comprises a large part of the germinating seed and is a nutrient storage organ that supplies nutrients to the growing embryo during germination. The mature cells that form the endosperm are packed with storage proteins and starch granules but are not living [28]. Therefore, there are two possible reasons that the relative GAD activity was observed in the endosperm. First, the GAD proteins stored in the endosperm might have been activated. Alternatively, the GAD enzymes might have been supplied from the scutellum or aleurone layer of the GAD seeds. For example, amylase, a starch-degrading enzyme, is supplied to the endosperm from the aleurone layer [28]. The results of this study are the first to suggest that GAD proteins may be supplied to the endosperm from other tissues. However, further studies, incorporating other techniques to track expressed proteins are required to verify this fact.

In this experiment, differences were observed in the mean activity when comparing embryos with and without salinity stress at different times after water uptake treatment (Figure S2). However, when comparing the embryo sizes at 24, 36, and 48 h (Figure 4), differences were observed. This is thought to be due to differences in water absorption and growth speed caused by salinity stress [17,18]. In contrast, no particular difference was observed in the size of embryos after 12 h of germination treatment. However, clear differences were apparent in the distribution of relative GAD activity throughout the embryo and seed coat (Figure 4). Therefore, a detailed investigation of GAD activity was conducted in the embryo and throughout the seed coat of germinated barley seeds after 12 h of imbibition treatment.

### 3.4. Visualization of Relative GAD Activity in the Embryo and Periphery of Seed Coat

**Figure 5:**
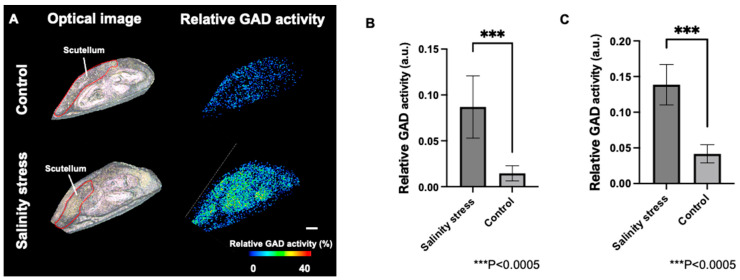
(**A**) Optical images and relative glutamate decarboxylase (GAD) activity distributions in barley seeds embryo with or without salinity stress. Scale bar is 200 μm. (**B**) Comparison of the GAD activity of barley embryo. (**C**) Comparison of the GAD activity of barley seed scutellum.

For a detailed investigation of the distribution of GAD activity in the embryo and pericarp of germinated seeds after 12 h of germination treatment, only the hypocotyl of germinated barley seeds was analyzed at high resolution (laser exposure interval: 15 μm; Figure 5A). A comparison of seed embryos exposed to salinity stress and control seed embryos by ROI analysis revealed that the relative GAD activity was significantly higher in seed embryos exposed to salinity stress (Figure 5B). Furthermore, when the scutellum of the plant seeds was investigated, the activity was significantly higher in seed embryos exposed to salinity stress than in the control (Figure 5C). The reproducibility of these results was confirmed (Figure S3). The scutellum of plant seeds is located at the interface between the plant embryo and endosperm, and is an important component involved in the secretion of enzymes for starch degradation and the absorption of degradation products [28]. In germinated rice seeds artificially treated with an abscisic acid analog, abscisic acid was shown to induce the expansion of scutellum tissue and the expression of polysaccharide metabolic enzymes and proteins in the tissue [29]. As abscisic acid is a phytohormone that plays an important role in various stress responses in plants, these results indicate that abscisic acid may be an important tissue involved in the salinity stress responses with increased GAD activity in the scutellum.

**Figure 6:**
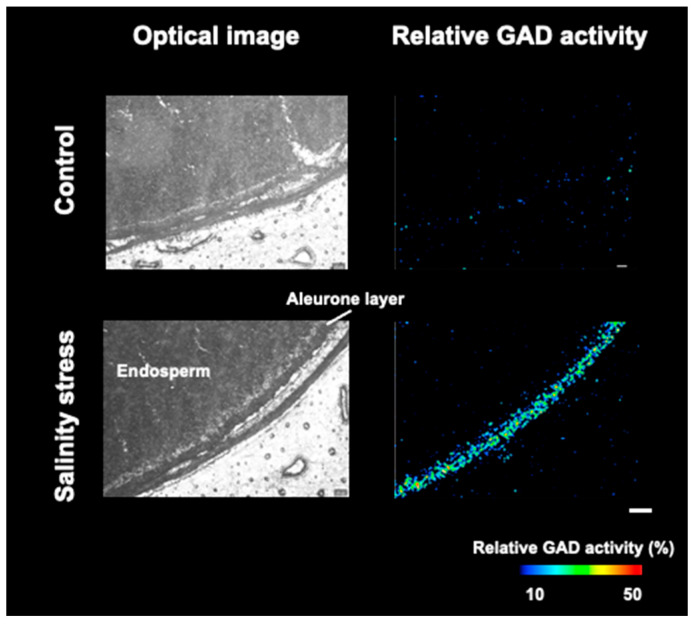
Optical images and relative glutamate decarboxylase (GAD) activity distributions in the area throughout the seed coat of germinated barley seeds with or without salinity stress. Scale bar is 200 μm.

In addition, the relative GAD activity in the area throughout the seed coat of germinated barley seeds exposed to salinity stress was compared to that of control seeds. Optical microscopy images showed no structural differences in the aleurone layer due to salinity stress, but high relative GAD activity was detected in the aleurone layer (Figure 6). Furthermore, the reproducibility of this phenomenon was confirmed (Figure S4). The aleurone layer, the cell layer between the seed coat and endosperm, is an important layer that contains many proteins involved in germination [28]. Immunohistochemistry showed that the gene expression of NADPH oxidase increased in the aleurone layer owing to imbibition during germination [30]. This suggests that the aleurone layer, as well as the embryo, is an important tissue involved in the salinity stress response through GAD and antioxidant enzymes.

## 4. Conclusions

In this study, MSI was successfully coupled with enzyme histochemistry to visualize the relative GAD activity during the germination of barley seeds. This technique was further applied to visualize the GAD activity in germinating barley seeds exposed to salinity stress, and LC/MS analysis revealed that salinity stress increased the GAD activity in germinated barley seeds. Visual localization of enzyme activity using MSI revealed that the increase in GAD activity was predominantly due to an increase in relative GAD activity in the seed embryo. Furthermore, we detected high GAD activity in the aleurone layer, the surface tissue of the seed, which is typically difficult to separate from the scutellum and measure using extraction analysis. This method, in combination with other imaging techniques, such as immunohistochemistry and in situ hybridization, has the potential to clarify the role of GABA shunts, including GAD enzyme responses, in barley seeds under stress. The results are expected to elucidate how GAD contributes to the germination process and salinity stress in plants, and to elucidate some of the mechanisms for salinity stress tolerance by plants. Eventually, this may lead to the development of high-salt stress tolerant varieties. Furthermore, the regulation of GAD activity in response to stress in seeds during germination may also affect GABA biosynthesis, and since MSI is a powerful tool for visualizing endogenous metabolites, it is important to simultaneously visualize the distribution of endogenous GABA and localization of GAD enzyme activity during seed germination and compare the distributions of GABA and GAD enzyme activity can be clarified. If these results can provide tissue-specific information related to high GABA production, they may contribute to the development of high GABA-containing varieties.

## Data Availability

The data presented in this study are available in the main article and the Supplementary Materials.

## Supplementary Materials

The following supporting information can be downloaded at: https://www.mdpi.com/article/10.3390/metabo12121262/s1, Figure S1: Comparison of relative GAD activity in barley germinated seeds at 12, 36, and 48 h after germination treatment by LC-MS analysis; Figure S2: Comparison of relative GAD activity in barley germinated seed embryos and endosperm after 12, 24, 36, and 48 h of germination treatment; Figure S3: The reproducibility confirmation of Figure 5 results. (A) Optical images and relative glutamate decarboxylase (GAD) activity distributions in barley seeds embryo with or without salinity stress. Scale bar is 200 μm. (B) Comparison of the GAD activity of barley embryo. (C) Comparison of the GAD activity of barley seed scutellum. Figure S4: The reproducibility confirmation of Figure 6 results. Optical images and relative glutamate decarboxylase (GAD) activity distributions in barley seeds embryo with or without salinity stress. Scale bar is 200 μm.
