# Supplementary material for: Visualization of Glutamate Decarboxylase Activity in Barley Seeds under Salinity Stress Using Mass Microscope
Source: Metabolites. 2022 Dec 14;12(12):1262. doi: 10.3390/metabo12121262 (PMC9786171; doi:10.3390/metabo12121262)
Supplement: Supplementary file 1 [file metabolites-12-01262-s001.zip › metabolites-2095474-supplementary.pdf]

Supplementary Figures

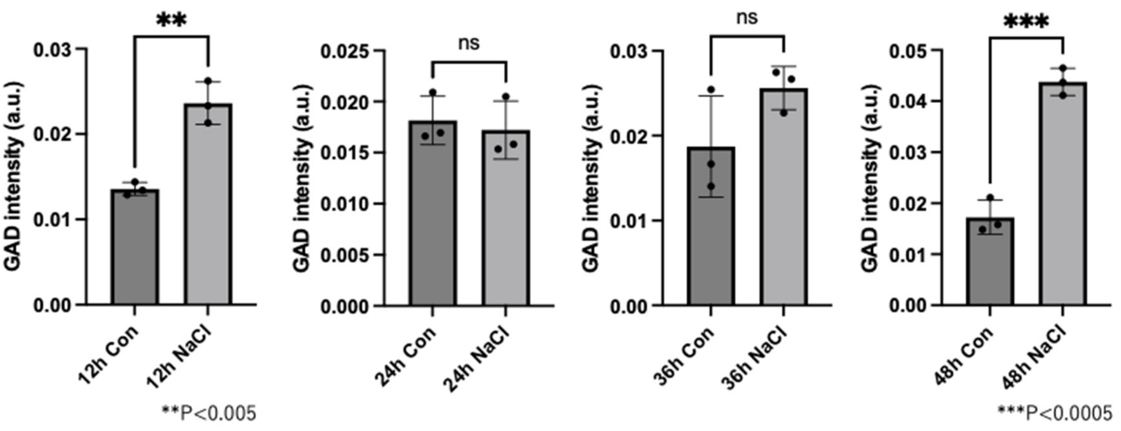

Figure S1 Comparison of relative GAD activity in barley germinated seeds at 12, 36, and 48 hours after germination treatment by LC-MS analysis

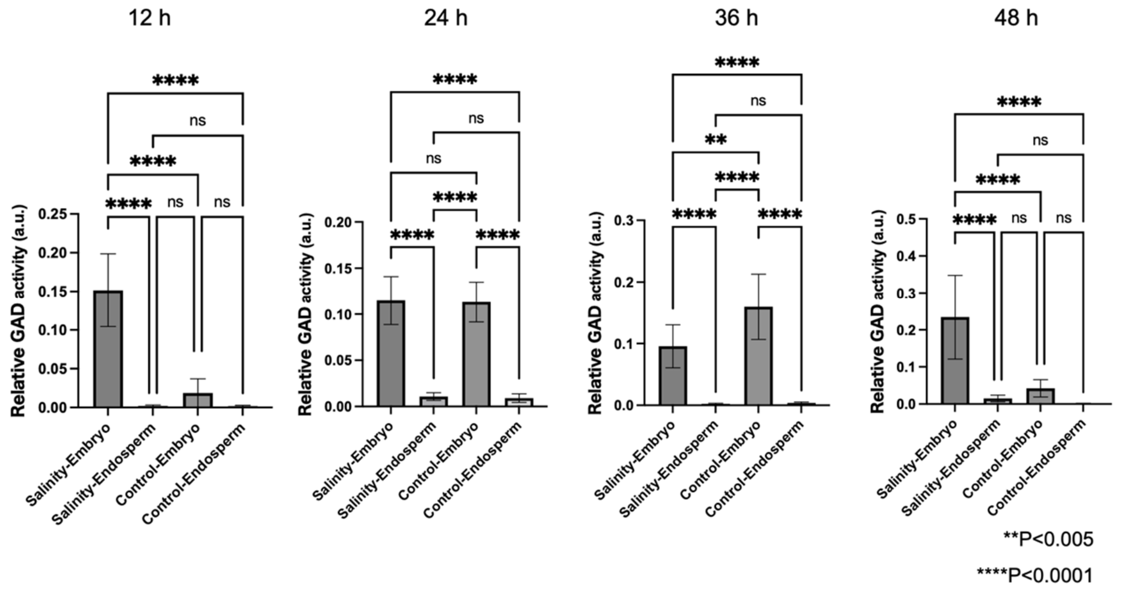

Figure S2. Comparison of relative GAD activity in barley germinated seed embryos and endosperm after 12, 24, 36, and 48 hours of germination treatment

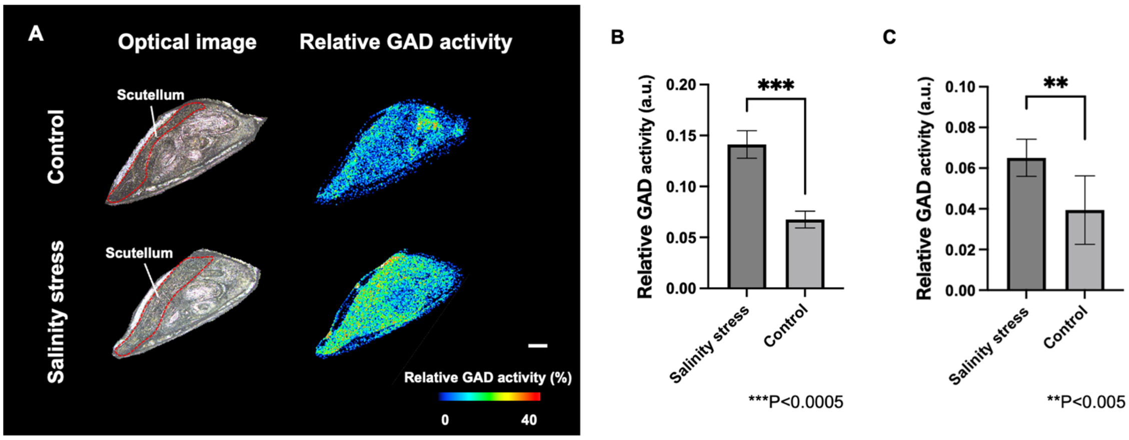

Figure S3. The reproducibility confirmation of Figure 5 results. (A) Optical images and relative glutamate decarboxylase (GAD) activity distributions in barley seeds embryo with or without salinity stress. Scale bar is 200 µm. (B) Comparison of the GAD activity of barley embryo. (C) Comparison of the GAD activity of barley seed scutellum.

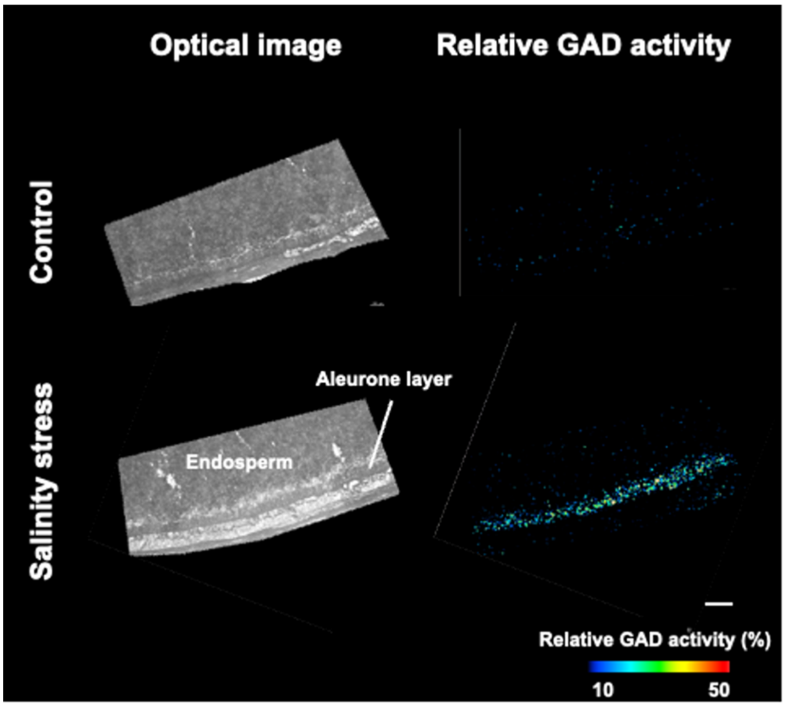

Figure S4 The reproducibility confirmation of Figure 6 results. Optical images and relative glutamate decarboxylase (GAD) activity distributions in barley seeds embryo with or without salinity stress. Scale bar is 200 μm.
